# An assessment of irrigated rice production energy efficiency and environmental footprint with in-field and off-field rice straw management practices

**DOI:** 10.1038/s41598-019-53072-x

**Published:** 2019-11-15

**Authors:** Nguyen-Van-Hung, Bjoern Ole Sander, James Quilty, Carlito Balingbing, Angeli Grace Castalone, Ryan Romasanta, Ma Carmelita R. Alberto, Joseph M. Sandro, Craig Jamieson, Martin Gummert

**Affiliations:** 1 International Rice Research Institute, Los Baños, Laguna 4031 Philippines; 2 Australian Center for International Agricultural Research, Canberra, ACT 2601 Australia; 3 Straw Innovations Inc., San Francisco, Victoria, Laguna 4011 Philippines

**Keywords:** Environmental impact, Climate-change ecology, Biodiversity

## Abstract

The research provided scientific evidences for improved rice straw management. Rice cultivation with in-field burning of rice straw is the worst option with the lowest energy efficiency and highest air pollution emission. This article comprises a comparative assessment of energy efficiency and the environmental footprint of rice production using four different rice straw management scenarios, namely, straw retained, straw burned, partial straw removal, and complete straw removal. Paddy yield, grain quality, and energy balance were assessed for two seasons while greenhouse gas emissions (GHGE) were measured weekly starting from land preparation through to the cropping and fallow period. Despite the added energy requirements in straw collection and transport, the use of collected rice straw for mushroom production can increase the net energy obtained from rice production systems by 10–15% compared to burning straw in the field. Partial and complete removal of rice straw reduces GHGE by 30% and 40% compared to complete straw retention, respectively.

## Introduction

Asia contributes about 670 million tons of rice to world production annually, which is approximately 90% of global production^1^. Correspondingly, in Asia a similar amount of rice straw is generated, much of which is burned in the field as a waste product after harvest. The practice of in-field straw burning results in greenhouse gas emissions (GHGE) of 0.7–4.51 g of CH_4_ and 0.019–0.069 g of N_2_O per kg rice straw burnt^2–4^. In addition to environmental impacts, emission from in-field rice straw burning can have serious negative consequences for human health as a result of the formation of suspended particulate matter (PM_2.5_ and PM_10_) in the air and the production of toxic gases^5–7^.

To avoid the negative impacts and reduce the environmental footprint of rice production, a range of rice straw management options are currently being developed and in some cases adopted in various Asian countries. Rice straw management and use vary. For example, in the Mekong Delta of Vietnam about 20–30% of rice straw is left in the field after harvesting, 50–60% is burned in the field, 10% is used for mushroom cultivation, and the remaining straw is used for animal feed or other purposes^8^. Gathering rice straw for energy production is a possible solution that can bring financial benefit to farmers, as well as reducing the environmental footprint of rice farming and preventing negative impacts of in-field burning. According to Gadde *et al*.^9^, the annual energy potential of rice straw produced in India, Thailand, and the Philippines, as a renewable fuel, is 312, 238, and 142 petajoule (PJ), respectively, at 100% collection efficiency, assuming that all harvested straw was used for energy production.

In intensive rice systems, with two or three crops per year, the large amounts of rice residues produced can impede land preparation, crop establishment, and early crop growth if they are left in the field. Long-term research at the International Rice Research Institute in the Philippines has shown that with careful and effective crop, soil, and water management all straw can be removed from flooded rice fields after harvest without reducing the levels of soil organic matter or soil fertility^10,11^.

Life cycle assessment (LCA) of rice production was reported in recent studies. Amarante *et al*.^12^ reported that using rice straw for bioenergy production through anaerobic digestion to be used as alternative or substitute to diesel fuel for transportation purposes has the lowest environmental impact as compared to other alternatives such as soil incorporation of rice straw. Soam *et al*.^13^ conducted a LCA study on cellulosic production from rice straw in India and reported the major benefits of GHGE and energy when using biomass to generate electricity resulting to displacement of coal-based electricity. The LCA on environmental footprint of rice production was also presented in other publications such as Blengini and Busto^14^; Thanawong *et al*.^15^; and Brodta *et al*.^16^.

However, these studies have not considered the various straw management practices utilized by farmers. To identify the best practices of rice straw management for irrigated rice production, within this study, we conducted a comparative analysis on energy balance and environmental footprint of rice production under contrasting rice straw management scenarios using an LCA approach and based on a field-experiment at the International Rice Research Institute in the Philippines. The knowledge gained from this research will illustrate the potential environmental and economic consequences of contrasting rice straw management options. While this study aims to identify the impact of contrasting straw management options on the energy balance and productivity of rice farming it does not aim to identify the total scope of agronomic changes that may be required to optimize these contrasting straw management scenarios.

## Materials and Methods

### Site description and experimental design

**Figure 1:**
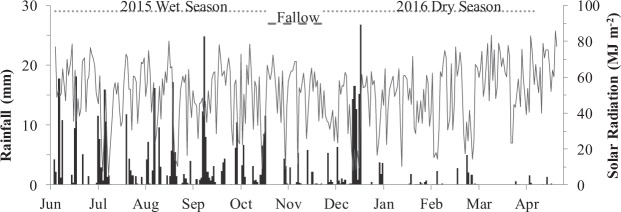
Rainfall and solar radiation data collected from the IRRI dryland weather station for the duration of the experimental period of the current study.

Two consecutive rice crops were produced in an experimental field between June 2015 and May 2016 to quantify the energy balance and environmental footprint of four contrasting rice straw management scenarios at the International Rice Research Institute (IRRI) research farm in Laguna, Philippines (14.148° N, 121.267° E) at an elevation of 27 meters. During the 2015 wet season (2015WS) crop (June 2015–October 2015) the total rainfall and solar radiation were 1207 millimeter (mm) and 2076 megajoule (MJ) m^−2^, respectively (Fig. 1). During the 2016 dry season (2016DS) (December 2015–May 2016) the total rainfall was 454 mm, while total solar radiation was 2016 MJ m^−2^.

Four rice straw management scenarios which are considered treatments were implemented in a completely randomized design using plots of 500 m^2^ with three replications. The treatments were classified as - all straw retained in the field after harvest (SRt), straw burning after harvest (SB), partial straw removal (PSRm) where about 60% rice straw was removed from the field after harvest and complete straw removal (CSRm) where both stubble and straw were removed from the field after harvest. The treatments were imposed at the start of the 2015WS after a uniform rice crop was harvested from the experimental field. LCA was undertaken on data collected from this field experiment in conjunction with an investigation of greenhouse gas emissions under contrasting straw management scenarios^4^.

The cropping schedule and agronomic practices used in this study were representative of common irrigated rice production practices in many rice growing regions of Southeast Asia (SEA). Each season during land preparation the primary tillage was undertaken with a disc plow under dry soil conditions using a four-wheel tractor, while the secondary cultivation, in the form of puddling and harrowing, was done under saturated soil condition with a two-wheel hand tractor. A final leveling before crop establishment was undertaken using a two-wheel tractor pulling a wooden plank approximately 3 meters wide. Fourteen day-old seedlings were used and established by manual transplanting in a 20 centimeter (cm) by 20 cm spacing. The rice variety grown each season was National Seed Industry Council (NSIC) Rc18 which is a popular variety in the Philippines. Transplanting was undertaken on the 18^th^ June 2015 for the wet season crop and on the 22^nd^ December 2015 for the dry season.

Fertilizer management in this experiment was uniform across all treatments with details of timing and rate provided in Table 1. The amount of fertilizer applied for both seasons was determined using the site specific nutrient management software which is the IRRI Rice Crop Manager. The fertilizer was a complete fertilizer (14-14-14), which was applied each season at 7–10 days after transplanting (DAT). The succeeding two splits of nitrogen were applied as Urea (46-0-0) at maximum tillering (28–30 DAT) and at panicle initiation (42–45 DAT). All fertilizers were applied using a Polaro fertilizer spreader manufactured by Lehner Agrar GmbH.

**Table 1:** Operations and agricultural inputs of rice production on the IRRI farm during the 2015 wet and 2016 dry seasons.

Processes	Agricultural inputs			Operation	Days before and after transplanting
	Materials	Quantity (kg ha^−1^)			
		2015WS	2016DS		
Plowing	—			Rotovator-4WT 35 hp	−20
Puddling	—			Hydrotiller-2WT 10 hp	−10
Harrowing	—			Powertiller-2WT 6.5 hp	−3
Leveling	—			Wooden plank-2WT 6.5 hp	−2
Seedling preparation	Seeds	20	20	Manual	−14
Transplanting	—			Manual	0
Fertilizer application				Spreader-2WT 5.0 hp	
	N	106	145		7
	P_2_O_5_	32	35		28–29
	K_2_O	32	35		44–48
Herbicide application	Pretilachlor	1.5 (a.i)	1.5 (a.i)	Manual	7
Harvesting (2015WS)				Manual and Thresher 21 hp	117
Harvesting (2016DS)				Combine harvest 67 hp	112

a. i. = active ingredient, 4WT = four-wheel tractor, 2WT = two-wheel tractor, hp = horse power.

Weeds were managed through the application of a pre-emergence herbicide with active ingredient (a.i) Pretilachlor, which was tank-mixed with molluscicide (a.i. Niclosamide) to control Golden Apple Snail (*Pomacea canaliculata*). The tank mix was applied immediately after transplanting. In addition to the application of the molluscicide, from 0 to 7 days after transplanting (DAT) the soil in the experimental field was kept saturated but without any standing water to reduce the risk of damage caused by Golden Apple Snail. Hand weeding was also conducted twice during each season to prevent and control weeds. In the 2015WS the irrigation supply was managed based on a conventional method of continuous flooding. Water was maintained at a depth of 3–5 cm from 14 days after transplanting until 10 days prior to harvest when the field was drained to encourage uniform maturity of rice grains and to enable harvesting activities to be conducted under dry soil conditions. In the dry season, mild water stress was experienced by the crop between tillering and flowering on a number of occasions, due to the limited availability of irrigation water. However, when water was available the field was kept flooded at 3–5 cm of standing water and a similar end of season drainage event was implemented as was achieved during the wet season.

### Soil sampling and analysis

To quantify any impacts of the straw management scenarios on soil chemical conditions, soil samples were collected from each experimental plot prior to land preparation at the start of the 2015WS and after harvest of the 2016DS crop. Composite soil samples of approximately 500 gram (g) dry weight were collected randomly from 0–15 cm using a soil auger with a diameter of 5 cm from within each experimental plot. The soil samples were air dried at 35–40 Celsius (°C) and then ground to pass a 2 mm sieve to remove any gravel before analysis. All soil analysis was conducted by the Analytical Services Laboratory at IRRI^17^. Quantification of cation-exchange capacity (CEC) was conducted using the indophenol blue method. Particle size distribution was determined using the hydrometer method with Calgon as the dispersing agent. Available potassium (K) was determined using ammonium acetate extraction with the extract being analyzed via flame emission spectrometry. Total soil carbon (C) and nitrogen (N) were quantified via combustion coupled with a gas chromatograph and thermal conductivity detector. Soil pH was determined in a 1:1 soil-water solution.

### Quantification of grain yield and total biomass

Straw biomass samples were collected immediately prior to harvest using three 1 meter (m) × 1 m sampling frames randomly placed in each plot with all above-ground biomass collected. Grain yield was determined from the total harvested yield from each experimental plot. The threshed paddy and the rice straw yield were weighed and recorded at fresh weight, and at dry weight (MC = 0) for both yield and biomass. The moisture content of the paddy was determined by the drying oven method^18^. Head rice recovery (HRR), a grain quality parameter that represents the percentage of whole grain recovery after milling, was calculated using Equation Eq. 1.

1 $$HRR\,( \% )=\frac{Weight\,of\,whole\,grains}{Weight\,of\,paddy\,samples}\times 100$$

To quantify HRR after threshing, three subsamples of at least 500 g of paddy were taken randomly from the grain harvested in each plot. After cleaning these samples using a Seedburo Paddy Blower, 250 g of filled grains were passed twice in the RISE 10” Rubber Roll Husker, then through a SATAKE Abrasive Whitener, and finally through a SATAKE Test Rice Grader. The whole grain recovered during milling was graded and weighed for the measurement of the HRR ratio.

### Life cycle assessment

**Figure 2:**
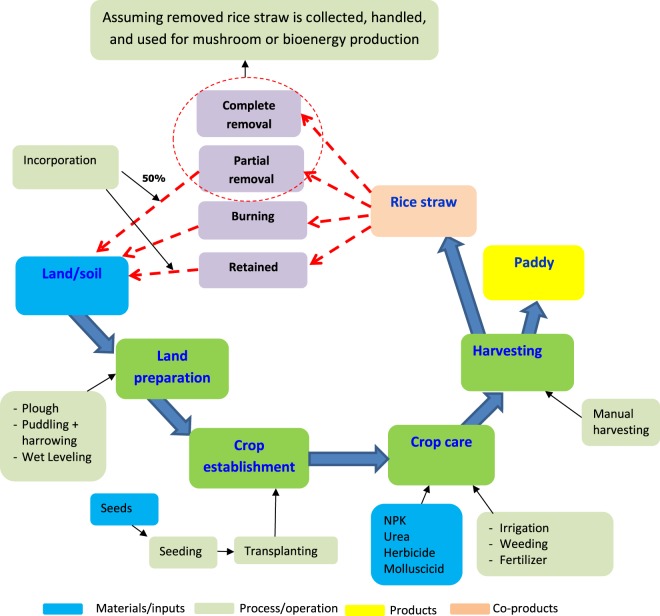
Research boundary of life cycle assessment in irrigated lowland rice production with different rice straw management options.

The life cycle assessment scope of this study is presented in Fig. 2 and its impact analysis was conducted using the SIMAPRO^19^ software. This study does not consider the effect of transport of agronomic inputs or the influence of irrigation source in the analysis, as generally there is limited opportunity for an individual farmer to manipulate these aspects of their farming practice. However, in the analysis of the energy balance and GHGE for rice straw, assumptions on the transport of this biomass and other factors in its utility outside of the field after harvest are included.

### Nutrient balance analysis

To calculate the balance of nutrient in straw incorporated into the soil, the nitrogen, phosphorous, and potassium content of straw retained in the field was measured from samples collected in the field immediately before in-field burning and incorporation. The laboratory result showed that rice straw contains 0.76–0.86% dry matter (dm) Nitrogen; 0.05–0.06% dm Phosphorus; and 1.30–1.54% dm Potassium.

### Energy balance analysis

An energy analysis of agronomic inputs and harvested outputs was undertaken over the duration of this study and both components were recorded and converted to an energy value using energy factors obtained from the available literature, as were the harvested outputs from the system. Net energy (NE) was calculated based on difference between the total input energy (IE) and output energy (OE) (Eq. 2).

2 $$IE={E}_{fuel}+{E}_{labor}+{E}_{agronomic}+{E}_{production}+{E}_{maintenance}$$

where *E*_*fuel*_, *E*_*labor*_, *E*_*agronomic*_, *E*_*production*_, and *E*_*maintenance*_ represent the energy consumed (MJ ha^−1^) converted from diesel consumption, labor, agronomic inputs (fertilizer and pesticide), production of machines and equipment, and machine maintenance, respectively.

The values obtained for harvested grain and rice straw were used to calculate the output energy of each straw scenario system. Pimentel and Pimentel^20^ quantified the energy content of rice grain as 15.2 MJ kg^−1^ dm, which is the value used in this study for all straw management scenarios. In contrast to grain as a human food source, the energy value of rice straw will vary depending on its treatment and utility. Within this research the energy value of rice straw was based on the following assumptions:

- Straw retained in the field (SRt and PSRm): energy accounted for N, P, and K contained in rice straw. These data were measured from straw sample analysis before incorporation into the soil.
- Straw burnt in the field (SB): energy value of this scenario was based on the N, P, K contents determined for the rice straw. However, losses of N, P, K in rice straw as a result of burning were assumed to be 100, 25, and 20%, respectively, as described by Dobermann and Fairhurst^21^.
- Straw removed from the field (PSRm and CSRm): the removed straw was accounted in the supply chain of mushroom production. Net energy of this production accounted for mushroom produced (economic based equivalent) and all the inputs (rice straw, water, power consumption, etc.) was about 3,500 MJ Mg of rice straw^22^.

The energy and GHGE conversion factors for agronomic inputs, processes, and products were presented in Table 2. The energy value and GHGE conversion factors of related materials were based on Ecoinvent database 3.0^5^, global warming potential 100 years (GWP-100a) of IPCC^23^ incorporated in SIMAPRO software^19^. Global warming factors – 100 years (GWP-100a) of CH_4_ and N_2_O are 30.5 and 265 kg carbon dioxide equivalent (CO_2_-eq). Production inventory data of energy and GHGE per unit of fertilizer chemicals refer to 1 kg N in urea with an N-content of 32%; 1 kg P_2_O_5_ in ammonium nitrate phosphate with a N-content of 8.4% and a P_2_O_5_-content of 52%; and 1 kg K_2_O in potassium chloride with a K_2_O-content of 60%. These data take into account the production activities including transports of raw materials and intermediate products but do not account for waste treatment of catalysts, coating and packaging. Similarly, energy and emission factors of herbicide are accounted for from their life cycle of production. Energy consumption and GHGE of machines were calculated based on 44.8 MJ L^−1^ of diesel^5^ accounting for production, transportation, and combustion in machinery. In addition 15 MJ L^−1^ was added for machine production^24^. Estimation of GHGE incurred from straw removal scenarios included emissions eminating from the straw collection process, transportation, and mushroom production. Estimation of emissions from straw collection were based on the study by Nguyen *et al*.^22^, which concluded that GHGE in this activity were between 60–165 kg CO_2_-eq Mg^−1^ of straw collected. Transportation of straw bales from the rice field to the site of mushroom production was assumed to be 10 km, which is equal to a GHGE of 5.78 kg CO_2_-eq Mg^−1^ of straw^5^. Emissions from the mushroom production process are estimated to be between 3.2 and 10.1 kg CO_2_-eq Mg^−1^ rice straw^25,26^.

**Table 2:** Energy and GHGE conversion factors of fuel, agronomic inputs, and products.

Parameters	Energy			GHGE		
	Unit	Value	Source	Unit	Value	Sources
Seeds	MJ kg^−1^	30.1	a, b	kgCO_2_-eq kg^−1^	1.12	a, b, n
Grain	MJ kg^−1^	15.2	c			
Diesel consumption	MJ L^−1^	44.8	a, b, d	kgCO_2_-eq MJ^−1^	0.08	a, b, n
Machine production	MJ L^−1^	15.6	d			
Nitrogen (N)	MJ kg^−1^	58.7	a, b, e	kgCO_2_-eq kg^−1^	5.68	a, b, n
P_2_O_5_	MJ kg^−1^	17.1	a, b, e	kgCO_2_-eq kg^−1^	1.09	a, b, n
K_2_O	MJ kg^−1^	8.83	a, b, e	kgCO_2_-eq kg^−1^	0.52	a, b, n
Herbicide	MJ kg^−1^	354	a, b, f, g	kgCO_2_-eq kg^−1^	23.3	a, b, n
Rice straw						
• collection and handling	MJ Mg^−1^	500	h, i, j	kgCO_2_-eq kg^−1^	0.12	h
• transportation (10 km)	MJ Mg^−1^	50	a, b	kgCO_2_-eq kg^−1^	0.006	a, b, h
• net energy and GHGE from mushroom production	MJ Mg^−1^	3500	h, i	kgCO_2_-eq kg^−1^	0.007	k
Manual labor			l, m			
• cultivation and drum seeding	MJ h^−1^	1.05				
• driving four-wheel tractor and combine harvester	MJ h^−1^	0.44				
• operating two –wheel tractor	MJ h^−1^	0.98				
• transplanting	MJ h^−1^	0.79				
• harvesting	MJ h^−1^	0.89				

a = ECOINVENT^5^; b = SIMAPRO^19^; c = Pimentel and Pimentel^20^; d = Dalgaard *et al*.^22^; e = Kool *et al*.^44^; f = Mudahar and Hignett^45^; g = Grassini and Cassman^45^; h = Nguyen *et al*.^23^; i = Nguyen *et al*.^40^; j = Nguyen *et al*.^26^; k = Ngo^25^; l = Quilty *et al*.^28^, m = Ainsworth *et al*.^27^; n = IPCC^24^.

Labor energy was calculated based on the metabolic equivalent of task (MET). Ainsworth *et al*.^27^ describes the MET as the ratio of human metabolic rate when performing an activity to the metabolic rate at rest. This ratio is converted to energy value as MJ h^−1^ using the method described by Quilty *et al*.^28^ with the assumption of an Asian human body weight of 54.4 kg^29^.

### Measurements and analyses of greenhouse gas emissions

The GHGE from the flooded soil were directly quantified in the field by Romasanta *et al*.^4^, while the emissions from fuel consumption and the indirect emissions from agronomic inputs were calculated using conversion factors presented in Table 2. Direct GHGE were measured *in-situ* in each experimental plot on a weekly basis during each of the two seasons. The total sampling period for GHGE each season was 175 days. GHGE sampling began prior to land preparation of the 2015WS crop, and continued through both cropping seasons and fallow periods after harvest. The GHGE samples were collected using the static chamber method as described by Sander *et al*.^30^. Emission factors from in-field rice straw burning used in this analysis are presented in Table 3. The emission factors for methane and nitrous oxide generated from straw burning are based on the results reported by Romasanta *et al*.^4^. Emissions were converted to Mg ha^−1^ rice straw at 14% MC based on the yield of rice straw in the experimental plots.

**Table 3:** Emission factors from rice straw burning.

Component	Emission factor		Environmental footprint factor		
	g kg^−1^ dw of straw	Sources	GWP-100a (kgCO_2_-eq kg^−1^ emission)	Human toxicity (kg1,4db-eq kg^−1^ emission)	Sources
CH_4_	4.51 (0.36)	a	30.5	—	h, i
N_2_O	0.069 (0.012)	a	265	—	
PM_2.5_	8.3	b	—	0.82	h, i
	12.9	c, d, e			
PM_10_	3.7	f, d, e, g	—	0.82	h, i
	9.4	b			

PM_2.5_ = Particulate matters (2.5 micrometers); PM_10_ = Particulate matters (10 micrometers);

*a* = *Romasanta et al*.^4^*; b* = *Nguyen et al*.^46^*; c* = *Hays et al*.^47^*; d* = *Ortiz de Zarate et al*.^48^*; e* = *Badarinath et al*.^49^*; f* = *Kadam et al*.^50^*; g* = *Gadde et al*.^6^*; h* = *ECOINVENT*^5^*; i* = *SIMAPRO*^19^*; dw* = *dry weight;*

The standard error of the mean is displayed in parentheses.

Table 3 also shows the other pollution factors from straw burning including particulate matters (PM_2.5_ and PM_10_). However, these pollution factors are simply used for discussing pollution and health problems that are not accounted for by GHGE. An indicator of pollution and health problems is represented by a human toxicity index with its unit of kg of 1,4-dichlorobenzene equivalent (kg 1,4db-eq). This can cause chronic and cancer effects with a risk threshold of 0.4 mg per kg of body per day or equivalent to 30 mg per person per day^31^.

### Statistical analysis and software

Analysis of Variance was used to evaluate the effects of the contrasting rice straw management scenarios on the measured production and environmental parameters using a least significant difference at *P* < *0.05* to compare mean values. The ANOVA was computed using the Statistical Tool for Agricultural Research (STAR) software developed by IRRI^32^. Energy balance analysis was based on the Cumulative Energy Demand 1.09 method^32^, and CO_2_ equivalent analysis was based on the GWP-100a of IPCC^23^. Conversion of agronomic inputs and fuel consumption to energy value was carried out using the Agri-Footprint, ECOINVENT 3, Industry Data 2.0 database^5^.

## Results

### Effects of straw treatments on soil chemical condition

No significant differences were identified in the measured soil properties between the experimental plots in the samples collected prior to land preparation for the 2015WS (Table 4). Similarly, there were no significant differences in the soil samples collected after harvest of the 2016DS. The mean value for all measured soil parameters, apart from soil pH, were found to increase in each straw treatment scenario between the initial soil samples collected before land preparation in the wet season of 2015 and the soil samples collected after harvest of the 2016DS crop. The mean soil pH values decreased, but not significantly, across all treatments between the initial and final soil samples. Significant differences (p-value < 0.05) were identified between the initial and final soil samples in the total soil C and CEC in both the PSRm and SRt treatments, and in available K in the SRt scenario (Table 4).

**Table 4:** Summary of results for soil parameters quantified from samples collected prior to the experiment being implemented in the 2015WS (Initial) and after the harvest of the 2016DS rice crop (Final) on the IRRI farm.

Treatments	Total C (%)^*^		Total N (%)^*^		Available K^*^		CEC (meq. 100 g^−1^)^*^		Soil pH^*^	
	Initial	Final	Initial	Final	Initial	Final	Initial	Final	Initial	Final
CSRm	1.29^c^	1.40^abc^	0.12^b^	0.13^ab^	1.49^ab^	1.52^ab^	29.8^ab^	31.2^ab^	7.10^a^	6.93^a^
PSRm	1.36^bc^	1.58^a^	0.13^ab^	0.15^a^	1.43^b^	1.57^ab^	28.9^b^	31.8^a^	6.90^a^	6.80^a^
SB	1.34^c^	1.44^abc^	0.12^b^	0.13^ab^	1.46^ab^	1.60^ab^	29.3^ab^	31.1^ab^	7.13^a^	6.93^a^
SRt	1.33^c^	1.56^ab^	0.12^b^	0.14^ab^	1.43^b^	1.61^a^	28.9^b^	31.6^a^	7.10^a^	6.93^a^

CSRm = Complete straw removed; PSRm = Partial straw removed; SB = Straw burned; SRt = Straw retained.

In a column, numbers followed by same letters are not significantly different by Turkey-Kramer test at 0.05 level.

### Rice grain and straw yield and head rice recovery

No significant differences in grain yield or head rice recovery were observed between any of the straw management scenarios in either of the seasons (Table 5). Across the four scenarios the rice yield in the wet season ranged from 4.9 to 5.2 Mg ha^−1^ in dry weight (dw). In the dry season the rice yield was lower than in the wet season, ranging from 3.5 to 3.8 Mg_dw_ ha^−1^. Similarly, HRR was higher in the grain produced in the wet season (62.5 to 64.1%) compared to the dry season (46.7 to 51.5%). The mean of straw yield at harvest was 2.54 Mg_dw_ ha^−1^. The harvest indexes (grain yield per total harvested biomass yield) were 0.66 and 0.52 for 2015WS and 2016DS, respectively. This factor, particularly for 2015WS was high mainly because of the short-plant variety (NSIC Rc18) with its plant length of about 1 meter. The mean amount of straw incorporated (SRt scenario) or burn (SB scenario) in the field were about 2.2 Mg ha^−1^ in dry weight, lower than the straw yield at harvest because of the mass loss from harvest to the straw treatments. Similarly, the mean amount of straw removed from the CSRm and PSRm scenarios was 2.2 and 0.9 Mg ha^−1^ in dry weight, respectively, lower than the straw yield at harvest because of accounting for the losses.

**Table 5:** The mean values for rice and straw yield, head rice recovery and amount straw incorporated and removed from each straw management scenario during 2015WS and 2016DS.

Treatments	Grain Yield (Mg_dw_ ha^−1^)	HRR (%)	Straw yield at harvest (Mg_dw_ ha^−1^)	Straw incorporated (1 month after harvest) (Mg_dw_ ha^−1^)	Straw removed (accounted for losses) (Mg_dw_ ha^−1^)
***2015WS***					
CSRm	5.10 (0.18)^a^	63.31 (1.22)^b^	2.60 (0.38)^c^	—	2.21 (0.23)
PSRm	5.21 (0.59)^a^	64.07 (2.05)^b^	2.41 (0.40)^c^	1.30 (0.27)	0.91 (0.27)
SB	4.95 (0.30)^a^	62.54 (2.91)^b^	2.48 (0.28) ^c^	2.21 (0.23)	—
SRt	4.93 (0.56)^a^	64.30 (0.94)^b^	2.67 (0.22) ^c^	2.21 (0.23)	—
***2016DS***					
CSRm	3.65 (0.58)^e^	49.04 (1.99)^f^	3.44 (0.26)^g^	—	2.23 (0.23)
PSRm	3.56 (0.46)^e^	46.68 (4.17)^f^	3.54 (0.89)^g^	1.37 (0.18)	0.86 (0.10)
SB	3.50 (0.36)^e^	51.31 (2.48)^f^	3.02 (0.57)^g^	2.23 (0.23)	—
SRt	3.79 (0.09)^e^	51.48 (1.62)^f^	3.24 (0.38)^g^	2.23 (0.23)	—

Mg_dw_ = Mega gram of rice straw in dry weight;HRR = Head rice recovery; CSRm = Complete straw removed; PSRm = Partial straw removed; SB = Straw burned; SRt = Straw retained.

The standard error of the mean is displayed in parentheses.

In a column, numbers followed by same letters are not significantly different by F-test Two-Sample for Variance at 0.05 level.

### Energy balance

The total fuel consumption in the 2015WS varied from 88 to 94 L ha^−1^ across the four straw management scenarios, which is not significantly different to the values recorded in the 2016DS in which the fuel consumption ranged from 87 to 89 L ha^−1^. In the 2015WS the total IE from manual labor activities for PSRm, SB, and SRt scenarios was 244–263 MJ ha^−1^ while for CSRm it was recorded at 361 MJ ha^−1^ with the additional labor for cutting the remained stubble after harvest. The IE values for manual labor during the 2015WS were more than double to that of the dry season for the PSRm, SB and SRt scenarios, and 50% higher in the CSRm scenario.

The total IE for rice production was approximately 14 and 16 GJ ha^−1^ for the wet and dry season, respectively (Table 6). The IE of the different scenarios in each season was similar because it did not include the energy for rice straw collection required in both the PSRm and CSRm scenarios. This energy for removing straw was accounted in the supply chain of mushroom production and generated an additionally net energy of 2.7–6.7 GJ ha^−1^.

**Table 6:** Input energy and output energy components and net energy (GJ ha^−1^) of the four straw management scenarios for the 2015 wet and 2016 dry seasons.

Items	Wet season				Dry season			
	CSRm	PSRm	SB	SRt	CSRm	PSRm	SB	SRt
***Inputs***								
Mechanized operations	5.46 (0.16)	5.37 (0.17)	5.63 (0.18)	5.66 (0.23)	5.37 (0.11)	5.27 (0.13)	5.32 (0.17)	5.35 (0.12)
Labor	0.36 (0.03)	0.24 (0.02)	0.24 (0.02)	0.24 (0.02)	0.24 (0.02)	0.14 (0.01)	0.14 (0.01)	0.14 (0.01)
Rice seeds	0.53	0.53	0.53	0.53	0.53	0.53	0.53	0.53
Fertilizer	7.05	7.05	7.05	7.05	9.42	9.42	9.42	9.42
Herbicide	0.51	0.51	0.51	0.51	0.51	0.51	0.51	0.51
*Total inputs*	*13.91 (0.19)*	*13.72 (0.19)*	*13.97 (0.20)*	*13.99 (0.25)*	*16.07 (0.13)*	*15.87 (0.14)*	*15.93 (0.18)*	*15.95 (0.13)*
***Outputs***								
Paddy	77.65 (3.19)	79.22 (10.36)	75.29 (5.33)	74.90 (9.90)	55.43 (10.32)	54.12 (8.05)	53.20 (6.36)	57.65 (1.66)
Rice straw (for mushroom)	6.62 (0.69)	2.72 (0.81)	—	—	6.70 (0.69)	2.59 (0.30)	—	—
Rice straw and ash incorporation	—	0.81 (0.12)	0.34 (0.04)	1.33 (0.14)	—	0.85 (0.85)	0.34 (0.34)	1.35 (1.35)
*Total outputs*	*84.27 (3.88)*	*82.75 (11.29)*	*75.63 (5.37)*	*76.24 (10.04)*	*62.13 (11.01)*	*57.56 (8.46)*	*53.54 (6.36)*	*59.00 (1.66)*
Net energy*	***70.36 (4.07)***^***a***^	***69.02 (11.48)***^***ab***^	***61.66 (5.57)***^***b***^	***62.24 (10.29)***^***ab***^	***46.01 (11.14)***^***c***^	***41.68 (8.60)***^***c***^	***37.62 (6.58)***^***c***^	***43.05 (1.93)***^***c***^

CSRm = Complete straw removed; PSRm = Partial straw removed; SB = Straw burned; SRt = Straw retained.

The standard error of the mean is displayed in parentheses;

*ANOVA for net energy: in this row, numbers followed by same letters are not significantly different by F-test Two-Sample for Variance at 0.05 level.

The total OE ranges were 76–84 GJ ha^−1^ and 54–62 GJ ha^−1^ for the wet and dry seasons, respectively. The higher OE achieved in the wet season was the result of the higher yield produced in this season. OE consists of energy obtained from paddy, using rice straw removed for mushroom production (for CSRm and PSRm), and incorporation of rice straw and ash retained in the field (for PSRm, SB, and SRt). Despite that, incorporation of all rice straw generated about 1.3 GJ ha^−1^, however the OE of SRt remained lower by 3–10% than that of PSRm and CSRm. The loss of the nutrients from burning of rice straw in the field in the SB scenario resulted in the OE of SB being 7–14% lower than that of PSRm and CSRm.

The net energy values ranged from 62 to 71 GJ ha^−1^ in the wet season and 38 to 46 GJ ha^−1^ in the dry season. Only the net energy of SB scenario in the wet season of 2015 was significantly different from that of the other scenarios. The net energy value of SB for wet and dry seasons recorded at 62 and 38 GJ ha^−1^, respectively, was the lowest due to the nutrient losses resulting from the in-field straw burning process.

### Environmental footprint

#### Greenhouse gas emissions

Table 7 shows the total annual GHGE of different scenarios accounting for both indirect and direct emissions. The total values for all scenarios ranged from 6.8 to 11.2 Mg CO_2_-eq ha^−1^ per year. Only the GHGE value of SRt scenario was significantly different from that of the other scenarios. The GHGE value of SRt at 11.2 Mg CO_2_-eq ha^−1^ year^−1^ was the highest due to the increased direct emissions from the soil resulting from straw incorporation that caused increased methane production. The order of annual GHGE from the four straw management scenarios from highest to lowest contributor is as follows - emissions coming directly from the soil during rice cultivation (63–84%); emissions from mechanized operations (9–15%); the embedded emissions in fertilizer production (6–11%); and emissions during in-field burning (11% for SB).

**Table 7:** Annual GHGE (kg CO_2_-eq ha^−1^) from the four rice straw management scenarios from the beginning of the 2015WS to the end of the 2016DS on the IRRI farm.

GHGE from	CSRm	PSRm	SB	SRt
Seeds	67	67	67	67
Fertilizer	1534	1534	1534	1534
Herbicide	66	66	66	66
Mechanized operations	864 (21.6)	849 (24.0)	875 (28.0)	879 (28.0)
Direct soil emission	3,739 (725)	4,875 (1,235)	4,168 (1,168)	8,671 (2,789)
In-field burning straw	—	—	694 (50.2)	—
Collection of rice straw (using round baler)	500 (65.8)	199 (18.5)	—	—
Transportation of rice straw (truck)	26	10	—	—
Mushroom production (silage and growing)	30	12	—	—
*Total*	*6,826 (813)*^*b*^	*7,612 (1,278)*^*b*^	*7,404 (1,247)*^*b*^	*11,217 (2,817)*^*a*^

CSRm = Complete straw removed; PSRm = Partial straw removed; SB = Straw burned; SRt = Straw retained. Global warming factors – 100 years (GWP-100a) of CH_4_ and N_2_O are 30.5 and 265 kg CO_2_-eq.

The standard error of the mean is displayed in parentheses;

*ANOVA for the Total GHGE: in this row, numbers followed by same letters are not significant different by F-test Two-Sample for Variance at 0.05 level.

#### Human toxicity

Rice production annually caused air pollution with a human toxicity index of 1150–1345 kg 1,4 DB-eq per ha, mainly constituting from 70–80% of fertilizer, 12–14% of pesticide, and 15% additionally caused by rice straw burning (SB scenario). The burning of straw generated pollutants such as 8–13 kg of PM_2.5_, and 4–10 kg of PM_10_. These total pollutants cause 150–200 kg of 1,4-dichlorobenzene equivalent per ha of rice production in a year with two-seasons of rice cropping, equaling to about 20–30 kg of 1,4-dichlorobenzene equivalent per ton of rice straw burned.

## Discussion

This study was just conducted under the condition of irrigated rice for one variety in the Philippines. However, the result would be an important evidence for promoting of more sustainable options of rice straw management rather than burning it in the field.

The changes in soil properties identified in this study may be the result of increased cropping intensity in the experimental field. For at least 10 years prior to the current study, the cropping schedule for this field had been one rice crop per year followed by a long fallow period of 6 months. The increases in total soil C and N, available K, and CEC are likely to be the result of increasing amounts of organic matter in the soil resulting from increased frequency and amount of above and below ground biomass production in the field. These results are similar to the findings of Thammasom *et al*.^33^, who reported an increase in soil CEC with the addition of 6.25 Mg ha^−1^ of rice straw after a single rice cropping season of 111 days duration. Our results potentially demonstrate the capacity of organic matter to provide exchange sites in the soil and improve soil C stocks. However, the longevity and extent of changes in soil properties resulting from the implementation of new agronomic management practices cannot be determined in a short-term experiment. More detailed investigations of the soil over a long-term period are required to draw conclusions on the longevity and sustainability of changes in soil condition.

While the results of this short-term study do not clearly demonstrate any influence of straw management on rice yields, a number of studies have concluded that incorporation of rice straw can have a positive influence on yields and soil fertility in rice production. Dobermann and Fairhurst^21^ claim that straw is often the only organic matter available in significant quantities to rice farmers, and others have concluded that the incorporation of rice straw into soil can help improve the fertility of soil and lead to improved yields^34,35^. Ponnamperuma^34^ demonstrated that the incorporation of rice straw resulted in an increase in the soil C and N content, and improvement in the availability of P, K and Silicon (Si) in the soil. Rice straw contains 0.5–0.8% N, 0.07–0.12% P, 1.2–1.7% K and 4–7% Sulfur (S), which are all essential nutrients for rice crop production^21^. However, other studies concluded that the incorporation of rice straw into anaerobic soils can result in the immobilization of N^36,37^, and has been attributed to rice yield decline^38^.

A study conducted by Pampolino^39^ demonstrated that the removal of rice straw does not necessarily result in a decline in soil organic matter in irrigated rice under continuous flooding. However, removal of rice straw can have a negative impact on the availability of soil nutrients if not carefully managed^21^. The maintenance of soil organic carbon (SOC) with complete straw removal is only shown for double cropped irrigated rice, it is not true for systems with one rice and one upland crop. In such systems the partial removal might be the more prudent option. A nutrient balance approach can be used to account for the impacts of straw management and determine fertilizer application rates required to sustain rice yields in systems where straw is removed^40^. Our study did not attempt to optimize the application rate of fertilizers for each individual scenario, but the nutrient balance differed between the contrasting straw management practices. Overall, considering to the air pollution and nutrient loss caused from rice straw burning and high GHGE caused from incorporation of rice straw in flooded fields, we would strongly recommend to promote the option of partial removal of rice straw using for mushroom production which can generate a value – added about 50–100 $US to a ha of rice production^41^ and also reduce GHGE by more than 30% compared with incorporation of all straw. In addition, spent straw after mushroom incorporation can be put back to the field to enhance soil nutrients, however on the other hand the incorporation of spent straw will increase GHGE that needs to be considered in the whole LCA.

**Figure 3:**
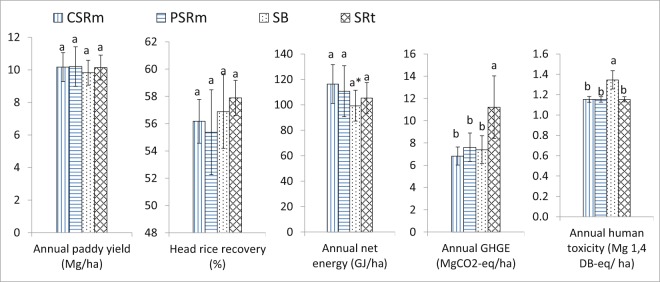
Comparison yield, head rice recovery, net energy balance, GHGE, and human toxicity of a 2-season irrigated lowland rice production with different rice straw management options. In a factor (i.e. paddy yield, head rice recovery, net energy balance, GHGE, and human toxicity), the columns followed by the same letters are not significantly different by the F-test Two-Sample for Variance at 0.05 level; *There was a significant difference between the net energy balance of CSRm and SB in 2015WS but not in 2016DS.

Comparison of yield, head rice recovery, net energy, GHGE, and human toxicity of irrigated lowland rice production with different rice straw management options is shown in Fig. 3.

The total input energy of rice production in our study closely agrees with that reported in research recently conducted in the Philippines^14,15,28^. The output energy values in the current study are slightly higher than those reported by Quilty *et al*.^28^, which is due to the energy values attributed to N, P, and K in rice straw, and the energy associated with straw utilization once it is removed from the field.

Utilization of rice straw removed from the field can increase the net energy obtained from rice production systems by 10–15% compared to burning straw in the field which demonstrated that the energy balance can be improved through the off-field use of rice straw in mushroom and bio-energy production. Our findings are consistent with previous reports^13,26,41^ and demonstrate the potential to add value to straw for farmers through off-field utility of this biomass. If off-field industries that use large volumes of rice straw can be successfully developed, then the frequency of in-field straw burning in many parts of SEA can be reduced. One aspect of off-field use of rice straw that could provide tangible solutions to reducing pollution by avoiding burning and reduce greenhouse gas emission (GHGE) is using rice straw for ethanol production as alternative fuel in place of gasoline^13^. More research is required to optimize rice production systems to ensure that the impact of straw removal from rice fields and the industries that utilize the straw are both sustainable.

Partial or complete removal of rice straw from the field reduces the GHGE by 30% and 40% compared to complete straw retention and incorporation, respectively which illustrated that removal of rice straw from the field for alternative uses has the added advantage of reducing methane emissions. Acharya^42^ also demonstrated that under anaerobic conditions the incorporation of rice straw into soil resulted in increased production of methane. This phenomenon means that irrigated rice production is a significant contributor to atmospheric greenhouse gases and global warming. However, the results of LCA studies investigating GHGE in rice production appear to be inconsistent, which may be due to a range of contributing factors. The total GHGE from rice production computed by Bordta *et al*.^16^ were similar to the current study, and used similar emission factors for the production of machines and agronomic inputs. In contrast, in the LCA studies reported by Hokazono and Hayashi^43^ conducted in Japan, and Blengini and Busto^14^ in Italy, the GHGE was more than double the current study. The difference is mainly attributed to differences in the emission factors (EF) used. For example, the EF of CH_4_ measured during direct field emission and then used for the LCA in our study is in the range of 4–22 g kg^−1^ of paddy. This is only 10–50% of the CH_4_ EF for the same category (48 g kg^−1^ of paddy) used in the other studies. Similarly, research undertaken in Thailand by Thanawong *et al*.^15^ used emission factors that were between 2 and 3 times higher than those used in our study.

The carbon footprint of agricultural production, in this case greenhouse gas emissions per rice yield, can be assessed through LCA and expressed as GHG Intensity (GHGI). The trend of the GHGIs followed what was reported for overall emissions because the rice yield did not significantly differ between treatments. The SRt treatment showed a GHGI of 1.02 kg CO_2_-eq kg^−1^ grain yield, the SB, PSRm and CSRm treatments follow with 0.54, 0.65 and 0.64 kg CO_2_-eq kg^−1^ grain yield, respectively.

Open-field burning of rice straw does not emit large amounts of GHGs. However, it generated the lowest net energy balance and could be assumed to have the lowest economic profit. In addition, the burning of straw generated pollutants such as 8–13 kg of PM_2.5_, and 4–10 kg of PM_10_ equaling to about 20–30 kg of 1,4-dichlorobenzene equivalent per ton of rice straw burned. To place this pollution figure in a specific context, the Human Toxic index was estimated for rice straw burning in the Mekong River Delta of Vietnam. If we assume that about 10% of the dry season crop, equal to a million tons of rice straw produced, are burned in the field over 15 days and affect a community of 10 million people, then about 90 mg 1,4db-eq per person per day is produced. This is three times higher than the risk threshold established by the EPA^31^.

## Conclusions

The research provided a scientific evidence for improved rice straw management. Rice cultivation with in-field burning rice straw is the worst option with lowest energy efficiency and highest air pollution emission. We demonstrated that despite the added energy requirements in straw collection and transport, the utilization of rice straw removed from the field for mushroom production can increase the net energy obtained from rice production systems by 10–15% compared to burning straw in the field. Additionally, partial or complete removal of rice straw from the field reduces the GHGE by 30% and 40% compared to complete straw retention and incorporation, respectively. Our findings were obtained from a two season experiment at a specific area in the Philippines, thus, it is difficult to extrapolate on a national scale. Additional data from other regions or long-term experiments should be gathered and more utilization options of rice straw such as for production of bio-char, compost, cattle fodder, bio-board or bio-plastic should be included in the LCA for a more comprehensive picture of the comparative environmental footprints of different straw management alternatives.

## Supplementary information

Dataset 1

## Data Availability

All data generated or analyzed during this study are included in this published article (and its Supplementary Information files).
